# The Relationship between Cancer and Paraoxonase 1

**DOI:** 10.3390/antiox11040697

**Published:** 2022-03-31

**Authors:** Irma Martha Medina-Díaz, Néstor Ponce-Ruíz, Aurora Elizabeth Rojas-García, José Francisco Zambrano-Zargoza, Yael Y. Bernal-Hernández, Cyndia Azucena González-Arias, Briscia S. Barrón-Vivanco, José Francisco Herrera-Moreno

**Affiliations:** 1 Laboratorio de Contaminación y Toxicología Ambiental, Secretaría de Investigación y Posgrado, Universidad Autónoma de Nayarit, Tepict 63000, Mexico; nestor.ponce@cinvestav.mx (N.P.-R.); erojas@uan.edu.mx (A.E.R.-G.); yael.bernal@uan.edu.mx (Y.Y.B.-H.); cyndia.gonzalez@uan.edu.mx (C.A.G.-A.); bbarron@uan.edu.mx (B.S.B.-V.); jfh2151@cumc.columbia.edu (J.F.H.-M.); 2 Unidad Académica de Ciencias Químico Biológicas y Farmacéuticas, Universidad Autónoma de Nayarit, Tepic 63000, Mexico; jzambran@uan.edu.mx

**Keywords:** cancer, PON1, inflammation, oxidative stress, PON1 activity, transcriptional factors

## Abstract

Extensive research has been carried out to understand and elucidate the mechanisms of paraoxonase 1 (PON1) in the development of diseases including cancer, cardiovascular diseases, neurological diseases, and inflammatory diseases. This review focuses on the relationship between PON1 and cancer. The data suggest that PON1, oxidative stress, chronic inflammation, and cancer are closely linked. Certainly, the gene expression of PON1 will remain challenging to study. Therefore, targeting PON1, redox-sensitive pathways, and transcription factors promise prevention and therapy in the development of several diseases, including cancer.

## 1. Introduction

Oxidative stress is an imbalance between the production and accumulation of free radicals and reactive metabolites, generally known as “reactive oxygen species” (ROS) in cells and tissues. ROS are typically generated as by-products of oxygen metabolism, which perform several physiological roles (i.e., cell signaling). Despite this, xenobiotics significantly increase ROS production, causing imbalances that lead to oxidative stress [1]. Under an imbalance sustained between free radicals and ROS production, significant damage to cell functioning, which consequently induces neoplastic transformation, occurs [2,3].

Elevated levels of ROS are thought to be oncogenic, damaging DNA, proteins, and lipids, and promoting genetic instability and tumorigenesis [4,5]. ROS also act as signaling molecules in cancer, contributing to abnormal cell growth, metastasis, resistance to apoptosis, and angiogenesis [3,6]. Additionally, increased levels of ROS are pro-tumorigenic, activating pro-survival signaling pathways, causing he loss of tumor suppressor gene function, and generating oncogenic mutations [6,7].

ROS are also responsible for the development of several diseases, including chronic inflammation, diabetes mellitus, and atherosclerosis [8,9,10]. Cancer onset in humans is a complex process requiring cellular and molecular alterations mediated by endogenous or exogenous triggers. Most significantly, oxidative DNA damage is responsible for cancer development [11,12,13]. Several studies have linked cancer initiation and progression to chronic inflammation and oxidative stress via increased DNA damage, cell proliferation, and genome instability [14,15,16].

Cancer is a disease of cellular mutation, proliferation, and aberrant cell growth, usually defined by at least three stages—initiation, promotion, and progression—that generally involve oxidative stress. The first stage of cancer (initiation) is defined as a stable, heritable change. This stage is irreversible and results in a mutational event produced by physical agents, chemicals, and ROS [17]. Additionally, initiating agents lead to genetic changes, including mutations, DNA damage, and structural alterations [18]. The second stage of carcinogenesis (promotion) is derived from either endogenous or exogenous stimuli of cell growth. ROS can alter the gene expression and induce the modification of the second messenger systems in this stage, increasing cell proliferation or inhibiting apoptosis and clonal expansion of initiated cells to produce a preneoplastic lesion. Finally, the third stage (progression) involves the conversion of benign preneoplastic lesions into neoplastic cancer [19].

Considerable evidence has demonstrated the role of antioxidants’ protective mechanisms based on enzymatic and nonenzymatic antioxidant molecules to link chronic inflammation and cancer [20,21]. In human cells, several genes and their products are involved with pro-oxidant and antioxidant functions for maintaining a balance in the oxidative homeostasis of a cell.

The most efficient enzymatic antioxidants involve superoxide dismutase (SOD), catalase (CAT), glutathione peroxidase (GPx), and glutathione reductase (GRx) [21,22,23]. Recently, paraoxonase (PON1), an antioxidant enzyme, has attracted significant interest as the protein responsible for most of the antioxidant properties of high-density lipoprotein (HDL) [24,25]. Studies have demonstrated that PON1 prevents the formation of oxidized low-density lipoprotein (LDL) and inactivates LDL-derived oxidized phospholipids once formed. Additionally, PON1 also retards the oxidation of phospholipids in HDL [24,26,27,28,29]. Furthermore, PON1 also scavenges carcinogenic lipid-soluble radicals [30,31]. This antioxidant enzyme also maintains the balance of antioxidants and oxidants [32,33], generally thought to contribute to cancer development [2,3].

## 2. Inflammatory Mechanism

Inflammation is a natural mechanism of the immune response to control infection, restore damaged tissue, or respond to stress control [34]. Traditionally, the signs of inflammation are described as redness, pain, heat, swelling, and disturbance of function [35]. Inflammation is beneficial in organisms as it represents the first barrier against infection or cell damage. It is a complex and controlled process where different innate and adaptive immune cells and molecules play different roles. However, failure in some of the mechanisms that regulate and resolve this process could lead to chronic inflammation and consequently degenerative diseases, such as cancer, diabetes, autoimmune diseases, and cardiovascular diseases [36].

**Figure 1:**
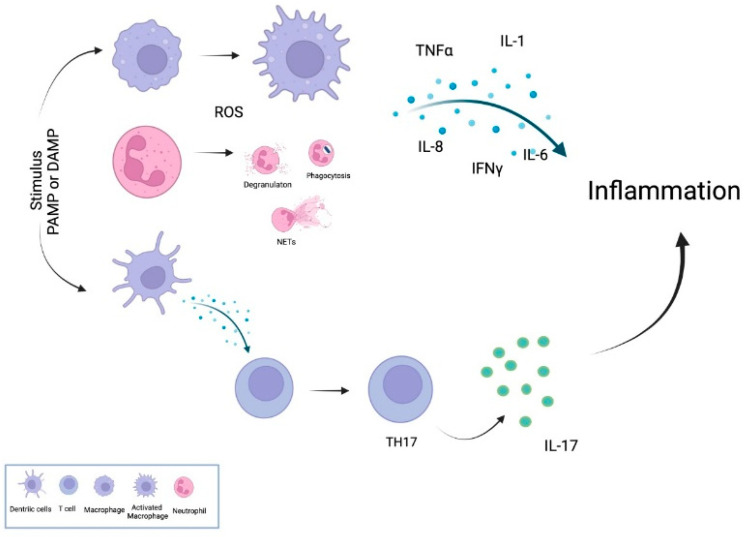
General mechanisms of inflammation. Innate immune cells (macrophages, dendritic cells, and neutrophils) stimulated by PAMPs or DAMPs, leading to their activation. Neutrophils display different mechanisms for eliminating pathogens. Dendritic cells are also involved in the differentiation of T cells to the TH17 phenotype. All these cells secrete different proinflammatory cytokines that promote the inflammatory process created with Bioreder.com (accessed on 28 March 2022).

As indicated in Figure 1, the inflammatory process is triggered by the recognition of the pathogen-associated molecular patterns (PAMP) or damage-associated molecular patterns (DAMP), both recognized by receptors expressed on the surface or cytosol of the immune cells at the site of the infection or lesion, named pattern-recognition receptors (PRR) [37]. These receptors transduce signals activating intracellular mediators, such as signal transducer and activator for transcription-3 (STAT3), nuclear factor kappa B (NF-kB), nuclear factor of activated T cells (NFAT), activator protein-1 (AP-1), and cAMP response element-binding protein, that induce the transcription of inflammatory genes, including cytokines and C-reactive protein (CRP) to restore structural and functional integrity [38]. The main proinflammatory cytokines secreted in response to a PAMP or DAMP recognition by activated immune cells are tumor necrosis factor-alpha (TNFα), interleukin (IL)-1β, IL-6, IL-8, and interferon-gamma (IFNγ) [34], among which TNFα is the primary inflammatory cytokine, due to their action on multiple cells [36]. Additionally, they trigger the recruitment of immune cells to infection sites. The primary inflammatory cells are neutrophils; however, macrophages [39], dendritic cells [40], and T-helper cells, in particular TH17 cells, also play an essential role in promoting and maintaining inflammation [41].

Neutrophils are the most abundant cells in innate immunity, usually recruited to infection or damaged sites by responding to chemoattracting signals [42]. Once in the inflammation site, neutrophils use their offensive mechanisms that include respiratory burst accompanied by ROS generation, degranulation (release of granules), the formation of neutrophil extracellular traps (NETs) [43,44,45], and the release of a broad range of mediators, including proinflammatory cytokines, alarmins, and proteases [44,46]. Additionally, neutrophils can communicate and interact with the antigen-presenting cells and T and B lymphocytes at the sites of inflammation [45].

However, macrophages are highly plastic cells that change their phenotype and respond to various environmental factors. Circulating monocytes, the precursors of macrophages, pass through the vascular endothelium to mature into macrophages in the peripheral tissues [47]. Macrophages form a heterogeneous cell population with different functions, divided into two subsets: the proinflammatory (M1) and the anti-inflammatory (M2) [47]. IFN-γ induces M1 macrophages-γ and bacterial lipopolysaccharide (LPS), thus producing various proinflammatory cytokines and high levels of oxidative metabolites necessary for host defense [48]. Meanwhile, the M2 macrophages are induced by IL-4 and IL-13, producing anti-inflammatory cytokines, including IL-10 [49], that promote angiogenesis and matrix remodeling [48]. Tissue macrophages are also responsible for secret inflammatory molecules that promote the recruitment of neutrophils from peripheral blood to the damaged or infected tissue [50]. Other innate cells, such as mast cells, are also important in the inflammatory process through the secretion of proinflammatory cytokines, including IL-1, TNFα, IL-18, and IL-33, that maintain the inflammation [51,52].

Neutrophils and macrophages produce ROS. Mitochondria mainly produce ROS, usually cleared by antioxidants, such as glutathione, vitamins C and E, and some enzymes such as catalase and peroxidase [53]. ROS induce the assembly and activation of inflammasome [54], a multiprotein complex responsible for activating the proinflammatory cytokines IL-1β and IL-8, in the context of the innate immune response [55]. In vitro studies have demonstrated that ROS induce the activation of the inflammasome, causing an increased secretion of IL-1β [56]. Additionally, the interaction of PRR with PAMP or DAMP activates transcription factor NFkB, which induces the production of IL-6, TNFα, and IFN-γ by neutrophils and macrophages [50,57].

ROS can cause oxidative damage of cellular macromolecules [57,58], which are inductors of the inflammatory process [50,59] by inducing the activation of the NFkB, the transcription factor responsible for the transcription and secretion of the proinflammatory cytokines IL-1β, IL-6, IL_22, and TNFα [57,60,61]. Moreover, the production of ROS is also involved in differentiation to TH17 cells [62]. Autophagia (a cellular process that removes damaged organelles) is a mediator of inflammation, with aberrant autophagia leading to chronic inflammation [63].

Dendritic cells (DCs) are antigen-presenting cells involved in priming and activating naïve T lymphocytes. They are also essential for sensing pathogens because they express PRR which recognizes PAMP or DAMP, leading to the secretion of IL-1β, TNFα, IL-6, and IL-12 [64,65].

The adaptative immune cells are also contributors to the inflammatory process. T-helper cells differentiate in effector cells after receiving signals from the T cell antigen receptor (TCR), cytokines, and costimulatory signals. T-helper effector subsets include TH1, TH2, TH9, TH17, and TH22 cells [66]. TH17 cells produce IL-17A, IL-17F, and IL-22 that protect the mucosa from bacterial and fungal infection [67], but have been implicated in inflammation [68] because IL-17 predominantly triggers the influx of neutrophils and tissue repair [69]. Additionally, dysregulated TH17 responses have been observed in inflammatory disorders, such as psoriasis, inflammatory bowel disease, rheumatoid arthritis, and type 1 diabetes [70], suggesting the essential role of these cells in inflammatory homeostasis.

Resolution of the inflammatory process involves macrophages, namely phagocytes, that detect the apoptotic cells and secrete IL-10 [71] and regulate B cells by secreting IL-10, IL-25, and TGFβ [72,73], which control the infiltration of inflammatory immune cells [71]. Although neutrophils are essential inflammatory cells, they generate several significant anti-inflammatory and pro-resolving lipid mediators, which program the resolution of inflammation [74]. Macrophages are also essential to resolve inflammation and avoid tissue damage because they are phagocytes that detect apoptotic cells and secrete anti-inflammatory molecules [50].

Although acute inflammation contributes to the protective host response, chronic diseases could result from uncontrolled inflammation and failure of immune cells to restore homeostasis. The imbalance between pro- and anti-inflammatory cytokines contributes to inefficient regulation of the inflammatory process [75]. There is increasing evidence of a close relationship between inflammation and cancer. Uncontrolled inflammation has been described as a critical mediator of tumorigenesis, involved in proliferation, angiogenesis, and metastasis [76], and mutagenesis of DNA in stem cells [77]. The relationship between inflammation and cancer has been studied over the years. In this context, inflammatory biomarkers, such as CRP, IL-6, IL-8, and TNFα, are associated with different types of cancer, such as breast and prostate [78]. In solid tumors, macrophages are the predominant immune cells correlated with high vessel density and tumor progression. M1 macrophages can kill tumor cells in vitro [47] or inhibit tumor growth [79]. In contrast, M2 macrophages facilitate tumor progression and invasion [47]. Inflammatory TH17 cells have been implicated in many types of cancer, including colorectal cancer, breast cancer, lymphoma, prostate cancer, gastric cancer, melanoma, hepatocellular carcinoma [80] and in cancer metastasis [81].

However, tumor cells also secrete some factors that serve as the immune response evasion mechanisms to avoid apoptosis; for example, the secretion of different molecules that promote regulatory T cells recruitment, polarize macrophages to an M2 phenotype [82], or promote the secretion of anti-inflammatory cytokines, such as IL-4, IL-10, IL-13, and TGFβ, that modulate the inflammatory response, which can also be involved in tumor progression [83,84]. However, tumor cells can also die by means of necrosis and release inflammatory signals into the surrounding microenvironment, resulting in the recruitment of inflammatory cells and promoting angiogenesis and tumor cell proliferation [85].

## 3. Genes Involved in Inflammatory Response

The inflammatory response is associated with genes involving balanced and regulated pro- and anti-inflammatory effects [34]. The protein products of these genes determine the outcome of inflammation. Most transcription factors that respond to inflammatory stimuli belong to families such as nuclear factor of the k light chain enhancer of B cells (NF-kB) [38], signal transducers and activators of transcription (STAT) [38], AP-1 [38], hypoxia-inducible factor-1α (HIF1-α) [86], NFAT [87], NF-E2 related factor-2 (Nrf2) [88], cyclooxygenase-2 (COX-2) gene [89], inducible nitric oxide synthase (iNOS) [90], inflammatory cytokines (TNF), IL-1β, IL-6 and chemokines [34]. Furthermore, the expression of microRNAs plays a role in oxidative stress-induced inflammation.

### 3.1. NF-κB Transcription

NF-κB transcription factors are implicated in regulating normal cellular and organismal processes, such as immune and inflammatory responses, developmental processes, cellular growth, and apoptosis [87].

Dysregulated NF-κB activity causes inflammation-related diseases and cancer. Two pathways activate NF-kB: the canonical pathway, which responds to an external stimulus involved in inflammation, immune response, cell proliferation, differentiation, and survival; and the second, the non-canonical pathway, associated with the development of cells in multiple layers [91]. For activating the canonical pathway, the signal-induced phosphorylation of IκB molecules by IkB kinase (IKK) complex (IKKα, IKKβ, and IKKγ) occurs in response to proinflammatory cytokines [92], thus leading to the K48-linked ubiquitination of IkBs and their degradation, then release of NF-kB from cytoplasmic inhibition [93,94]. As a result, NF-κB dimers translocate to the nucleus and induce the transcription of the target gene [93,95,96]. However, canonical NF-κB is activated in innate and adaptive immune cells by signals via innate PRRs, TCR, B-cell receptor, proinflammatory cytokine receptors, and others [95,97,98].

PRRs expressed in innate immune cells recognize microbial PAMPs or DAMPs, therefore inducing the expression of proinflammatory cytokines, such as TNF-α (tumor necrosis factor-alpha), IL-1β, IL-6, IL-8, IFN-I (type I interferons), chemokines, and anti-microbial proteins, which leads to the inflammatory response [87,99,100,101]. 

However, cell differentiating or developmental stimuli, such as BAFF, RNAKL, or lymphotoxin, activate the non-canonical NF-κB pathway to induce RelB/P52 dimer in the nucleus. In this pathway, ligands induce NF-κB, encouraging kinase (NIK) activation. NIK phosphorylates NF-κB2 protein leads to proteasomal processing of the NF-κB2 precursor protein p100 into the mature p52 subunit. Then, the p52 dimerizes with RelB to regulate a distinct class of genes [102]. 

### 3.2. Signal Transducers and Activators of Transcription

Some studies have suggested a role for STAT family proteins, especially STAT3, in inducing and maintaining a pro-carcinogenic inflammatory microenvironment. STAT3-mediated malignant property is associated with chronic inflammation. Cytokines, chemokines, and other mediators are essential for inducing and maintaining a cancer-promoting inflammatory environment, where STAT3 regulates their expression. Moreover, it has been suggested that NF-κB and STAT3 act as two major transcriptional factors linking inflammation with tumorigenesis and functionally interacting with each other [87]. Therefore, it has been suggested that understanding this interaction could promote better understanding for developing a more effective treatment against cancer, including its prevention.

### 3.3. Activator Protein 1

NF-κB and AP-1 are essential in regulating immune genes and inflammation progression. However, it has been reported that the anti-inflammatory mechanism involves the inhibitions of a transcription factor, such as NF-κB, STAT, MAP, and AP-1. AP-1 is a heterodimeric protein composed of proteins ATF and JDP, c-Jun, c-Fos, capable of modulating the expression of genes, such as *TNF-α*. Additionally, AP-1 is related to asthmatic inflammation, cardiovascular disease, cancer, and others [103,104].

### 3.4. Hypoxia-Inducible Transcription Factor

The hypoxia-inducible transcription factor (HIF) plays a fundamental role in metabolic changes, which conduct cellular adaptation to low oxygen accessibility through the transcription of genes possessing an HRE. Cellular HIF expression can be modulated by hypoxia, inflammation, cancer, among other factors [105,106]. Evidence in the literature showed that adaptive and innate immune systems are modulated by hypoxia and HIF activity. Furthermore, different key factors in the inflammation process induce HIF-1α protein, in normal oxygen conditions, such as ROS, TNFα, NF-κB (induced HIF-1α mRNA), LPS (by transcriptional and translational mechanisms), and LDL [105].

### 3.5. Cyclooxygenases

Cyclooxygenase is a key enzyme in synthesizing prostaglandins through the oxidation of arachidonic acid. Prostaglandins are related to inflammation, pain, the development of cancer, and some functions of homeostasis in various organs [105].

There are two isoforms of cyclooxygenases, COX-1 and COX-2, encoded by different genes, which are also located in different cell sites with different affinities for their substrate. COX-1 is expressed constitutively and plays a major role in the different homeostatic processes. In contrast, COX-2 has a constitutive expression in organs, such as the brain, kidney and an inducible enzyme. The metabolites of COX-2 are involved in inflammation and pain induction [107,108]. Additionally, COX-2 cDNA encodes a 70-kDa protein containing 604 amino acids. LPS could also induce COX-2 mRNA in human endothelial cells [109]. A high overexpression of COX-2 results in the high synthesis of PGE2 and increased cellular proliferation, which is key in cancer development [89].

The *COX-2* gene contains typical regions, such as those of early response genes, which permit fast upregulation when stimuli that trigger cytokines such as TNF interleukin-1 are present. Additionally, the COX-2 mRNA has labile sites encouraging degradation of the fast product and consequently decreasing gene expression when the stimulus is withdrawn. The *COX-2* upregulation gene is observed in cell types such as macrophages and endothelial cells, among others. However, glucocorticoids and other anti-inflammatory agents decrease the *COX-2* gene expression [107,109]. 

Although a large diversity of elements control the expression of COX-2, two important elements are b-catenin (directly by binding to the promoter or indirectly) and NF-kB. Additionally, the COX-2 mRNA is regulated at the post-transcriptional level through translation mechanisms and stability, mediated by AU-rich sequence elements. Several proteins play a crucial role in these events; among them are AUF1, HuR, CUGBP2, hnRNPA1 TIA1, TIAR, and TTP [89]. 

### 3.6. RNA-Based Processes

The data reported in the literature show the relevance of RNA-based processes in controlling inflammatory responses. Additionally, non-coding RNAs (ncRNAs) have been reported in inflammation regulation. In this regard, long non-coding RNAs can mediate the interaction between DNA and proteins or modulate the translation (through miRNAs or mRNAs) [110].

## 4. Proinflammatory Cytokines and PON1

The proinflammatory state is characterized by high circulating levels of proinflammatory markers, including IL-1β, IL-1 receptor antagonist protein (IL-1RN), IL-6, IL-8, IL-13, IL-18, CRP, IFNα, and IFNβ, transforming growth factor-β (TGFβ), TNF and its soluble receptors (TNF receptor superfamily members 1A and 1B), and serum amyloid A [111].

Higher circulating proinflammatory cytokine levels confer deleterious effects on the remote organs. Most comorbid conditions such as hypertension, diabetes, obesity, and dyslipidemia annotated with cardiovascular risk have chronic inflammation associated with elevated levels of proinflammatory cytokines, in particular, TNFα, showing a more significant impact on cardiac function and remodeling [112,113,114,115,116].

PON1 is recognized as an antioxidant enzyme that inhibits LDL oxidation. Furthermore, it is accepted as an enzyme with anti-inflammatory features that limit the release of proinflammatory mediators [117]. It is well-known that oxidized LDL (ox-LDL) promotes endothelial cell activation, dysfunction, death, causing onset and progression of atherosclerotic process [118,119]. Additionally, endothelial injury by oxLDL causes the expression of adhesion molecules and chemotactic cytokines, thereby stimulating the activation and migration of immune cells, thus exacerbating the inflammatory processes and, consequently, the formation of atherosclerotic plaque [120]. Noteworthily, the vascular protective role of PON1 is not limited to the action on oxLDLs; the HDL-associated enzymes also serve as an antioxidant shield against oxidative insult against immune cells in resident macrophages and endothelium [121]. Additionally, compelling studies show the ability of PON1-containing HDL to modulate monocyte chemotactic protein-1 (MCP-1) secretion from endothelial cells and to suppress the proinflammatory response of macrophages [122,123]. Thus, since PON1 inhibits the formation of oxLDL and, consequently, the release of proinflammatory cytokines, it is relevant to elucidate its role in inflammatory disease development.

Nevertheless, the activity of this enzyme is modulated under oxidative stress conditions [124]. Furthermore, PON1 has been implicated in the pathogenesis of several inflammatory diseases, including atherosclerosis, diabetes, and cancer. Since it is reported that the proinflammatory cytokines, such as IL-1β and TNFα, downregulate PON1 expression and secretion by liver cells, the lower levels of PON1 in serum can likely be a long-lasting inflammatory resultant condition [125]. Therefore, PON1 has attracted increasing interest since its role in this pathogenesis condition remains unclear regarding whether its status is a causative factor or biomarker for these diseases.

As mentioned above, studies have shown that hepatic PON1 is regulated in response to inflammatory cytokines. Treatment with lipopolysaccharide and oxidized LDL in animal models and human hepatocytes reduced the mRNA levels of PON1. This effect was similar in the presence of TNFα, IL-1β, and IL6 [126,127]. Moreover, the stimulation of a murine hepatic cell line with a mixture of cytokine (IL6, TNFα, IL-1β) increased serum amyloid A (SAA) expression in parallel with a decrease in *ApoA1* and *PON1* mRNA. There is a nuclear factor-κB (NF-κB) binding site on the promoter of SAA but not in ApoA1 or PON1 promoters. The inhibition of NF-κB decreases SAA mRNA and consequently increases the *ApoA1* and *PON1* mRNA levels [128]. The results suggest that cytokines both upregulate SAA and downregulate PON1 and ApoA1 via NF-κB. Activation of PPARα antagonizes NF-κB, leading to an attenuation of the inflammatory response. It is unclear whether PPARα interacts directly with the PON1 promoter or acts via an unidentified factor [128,129]. In contrast, the treatment of HepG2 cells with IL6 significantly increased the function and protein levels of PON1. Additionally, IL-6 might upregulate the expression of an anti-atherosclerosis molecule *PON1* gene at a transcriptional level through the AKT/IKK/NF-κB activation pathway in human hepatocyte-derived HepG2 cells [130]. PON1 and SAA are inversely related and may act as markers to assess HDL function in oxidative stress and inflammation [128,131]. Studies have assessed the effects of chronic inflammation, revealing that prolonged SAA secretion by the liver is associated with lower ApoA-I and PON1 activity [128,132].

Additionally, some studies in humans have shown a relationship between cytokines and low PON1 activity. For example, Kerekes et al. [133] observed in patients with rheumatoid arthritis (RA) that PON1 activity significantly correlated with serum levels of TNFα and interleukin-6 in the patients with RA, suggesting a possible relationship with RA disease activity. Additionally, Szczeklik et al. [134] found in patients with Crohn’s disease that the decrease in the levels of PON1 was correlated with the disease’s activity and reflects the intensification of inflammation and intensified lipid peroxidation.

Recently, PON1 has been suggested to protect cells from oxidation by inhibiting the synthesis of chemokine (C-C motif) ligand 2 (CCL2) and the subsequent inflammatory reaction. However, excessive production of ROS would inhibit PON1 and increase CCL2 production. This induces the migration and infiltration of immune cells in target tissues, disturbing normal metabolic function. This disruption involves pathways controlling cellular homeostasis and metabolically driven chronic inflammation, stating its relationship with the development of some diseases [27].

## 5. Structure of the *PON1* Gene and Polymorphism

The human PON1 is a circulating Ca^2+^-dependent A- esterase with a molecular mass of 43 kDa and 354 amino acids [135,136]. PON1 is one of the three isoforms of paraoxonase (PON 1, PON2, and PON3) located on the long arm of chromosome 7 (7q21.3) [137]. This enzyme is an aryldialkylphosphatase (EC 3.1.8.1) mainly synthesized by the liver, in lesser amounts in the kidney and colon, and then secreted into the bloodstream, where it is tightly bound to HDL particles [136,137,138]; however, it can also be transported from these particles to the cell membranes [25], especially those of epithelial and endothelial cells [139,140].

PON1 received its name from its ability to hydrolyze paraoxon (diethyl p-nitrophenyl phosphate), an active metabolite of the insecticide parathion [141,142,143,144]. Additionally, PON1 hydrolyzes several organophosphate xenobiotics, nerve agents, cyclic carbonates, glucuronide drugs, estrogen esters, and arylesters [136,138,145,146,147]. 

The original function of PON1 was in lactonase (LAC), since lipophilic lactones constitute its primary substrates, shown to hydrolyze over 30 different lactones (cyclic esters) [148,149,150]. This catalytic capacity enables PON1 to degrade lipid peroxides within the cell and lipoproteins in circulation [24,28,29]. However, PON1 has also been shown to be involved in arylesterase (ARE), phospholipase A2-like activity, estrogen-ester hydrolase, cyclic carbamate hydrolase, and possibly peroxidase-like activity [29,145,149]. 

Additionally, several physiological functions have been attributed to PON1, particularly its degradative capacity of lipid peroxides [151,152,153], as an antioxidant molecule, and in the control of inflammation [26,27]. PON1 protects against oxidative stress through the hydrolysis of active oxidized phospholipids, destroys lipid hydroperoxides and H_2_O_2_ (via its peroxidase-like activity), preserves HDL integrity and function, and prevents LDL oxidation and the cell membrane; it is thought to be atheroprotective [31]. Additionally, PON1 protects against postprandial oxidative stress, detoxifies homocysteine-thiolactone, inhibits cholesterol biosynthesis in macrophages, and increases its flux in similar cells, modulating lipid metabolism in adipose tissue. PON1 can also protect the organism from bacterial biofilm formation through its LAC activity, preventing infection by Gram-negative bacteria, such as *Pseudomonas* [20,136,153,154,155,156].

The expression and activity of PON1 have been associated with lung cancer, multiple myeloma, and papillary thyroid cancer [157,158,159]. Additionally, alterations in circulating PON1 levels and activity have been reported in several diseases involving oxidative stress and inflammation, including cardiovascular diseases, chronic renal failure, Alzheimer’s disease, metabolic syndrome, and chronic liver impairment [27,29].

### 5.1. PON1 Gene Structure

The *PON1* gene is located between q21.3 and q22.1 on the long arm of chromosome 7 in humans (chromosome 6 in mice) [136,137]. *PON1* gene size is approximately 26 kb, which comprises nine exons and an extra codon at position 106 (lysine) in exon 4 [137,160]. *PON1* appears to be a non-canonical polyadenylation signal sequence. The fourth intron (of eight) contains a CA repeat, the length of which is polymorphic, and allele lengths vary by up to four CA units [137,161].

The promoter sequence of the *PON1* gene does not contain a canonical TATA box in the 5′ UTR but is rich in GC sequences [137]. Furthermore, the *PON1* gene has 200 polymorphisms distributed between the promoter and its coding region, but most are not characterized. It has been described that the polymorphisms of the promoter impact the regulation and expression of the gene. However, the polymorphisms of the coding region have a more significant impact on its activity in serum. A potential role for the transcription factor specificity protein 1 (Sp1) was demonstrated by Deakin et al. [162]. Sp1 is a ubiquitous transcription factor implicated in expressing several genes. Therefore, it does not singly indicate what pathways regulate *PON1* expression. However, one set of transcription factors with which also act in tandem are the sterol-regulatory-element-binding proteins (SREBPs) [162]. Therefore, variations in promoters are physiologically relevant, showing significant differences in serum concentration and activity of PON1. More information will be addressed in the section on gene regulation and its polymorphisms.

*PON1* mRNA is expressed in the liver, with the PON1 glycoprotein consisting of 354 amino acids (molecular mass of 43-KDa). It retains its hydrophobic signal sequence in the N-terminal region (except for the initial methionine), which enables its association with HDL [163]. Furthermore, PON1 was localized almost exclusively in the densest HDL3b and 3c subfractions, containing apo A1 and clusterin [164]. Therefore, the dense HDL3 fractions are recognized most potently protective on a per-particle basis among all HDL subpopulations [164]. Furthermore, apolipoproteins form the basis for functional heterogeneity of HDL, as suggested in several previous studies.

An X-ray crystallography study has indicated the structure of PON1 as a 6-bladed propeller, with a lid covering the active site passage and containing two Ca^2+^, one essential for activity and interaction with the side chain oxygens (N224, N270, N168, D269, and E53) as an oxyanion that stabilizes the substrate and necessary for optimal hydrolytic activity. The second ion is essential for stability with its removal causing irreversible structural disruption of protein structure [165,166]. Therefore, this implicates gene expression as a major source of variations in serum PON activities and concentrations [167,168].

### 5.2. Single-Nucleotide Polymorphisms of the PON1 Gene

#### 5.2.1. Polymorphism in the Promoter Region

The *PON1* gene has nearly 200 SNPs. The main polymorphisms identified in the promoter region of the *PON1* gene are −909 (G or C), −832 (A or G), −162 (A or G), −126 (C or G), and −108 (C or T). −108C, −832A, −162A and −909G have higher levels of expression than −108T, −832G, −162G, and −909C. The literature that identified the positional changes reported these polymorphisms (−107 or −108, −160 or −162, −824 or −832, −907 or −909) due to the variations in the examined sequences. Despite the physiological relevance of the coding region polymorphisms, an association with highly significant regulatory region polymorphisms in PON activities has been described in the case of the −107C and −824A polymorphisms. The −107C and −824A variants have higher activity than the −107T and the −824G variants [168,169]. However, other studies revealed no significant contribution for the −824 and −907/−909 sites [162]. In the case of the position of the −909 polymorphism, it was shown to be in linkage disequilibrium with the other sites, having little or no independent effect on the level of PON1 activity in vivo [170]. It is believed that −108C/T polymorphism is the main contributor to variation of PON1 in serum, explaining approximately 23–24% of the total variation, while −162A/G, −909G/C, and −832A/G showed little or no effect on serum levels PON1 [162,170]. The −108C/T polymorphism was a consensus-binding site for the ubiquitous transcription factor Sp1. On the one hand, overexpression of Sp1 dramatically enhanced PON1 promoter activity. These data suggest that Sp1 acts as a positive regulator of *PON1* transcription, whereas mithramycin acts as an inhibitor [171]. The presence of T at position −108 interrupts the sequence that recognizes Sp1 by binding the factor, weaker in the presence of T than in C. However, this Sp1 was bound, indicating that this region only partially regulates the transcription of *PON1* [162]. The polymorphism at position −162 is located in a consensus-binding site for the NF-1 (nuclear factor-1). The presence of G at the position −162 disrupts the binding site’s sequence, resulting in lower gene expression [170,172]. Polymorphisms have been identified in the 3′-untranslated regions of the *PON1* gene; however, their significance is not yet studied. A significant increase in serum PON1 mass and activities between haplotype subgroups could be explained by a unit increase in the number of high-expresser variants of the −108 site (−108C) alone. Several studies have revealed that the polymorphisms at positions −126 [169] and −824 [170,173] do not seem to impact PON1 plasma concentration or activity, regardless of the allele present.

#### 5.2.2. Polymorphism in the Coding Region

Studies in the early 1990s led to the purification, cloning, and sequencing of human PON1 and the molecular characterization of its polymorphisms [174,175,176]. Additionally, the molecular structure of a recombinant PON1 protein has also been elucidated [163]. 

Human PON1 has nearly 200 SNPs [177,178]. Of these −909G/C, −162A/G, −108C/T polymorphisms located in the promoter region and L55M and Q192R in the coding region of PON1 are the most commonly studied SNPs (Table 1). The genetic variations of the *PON1* modify PON1 properties, and some SNPs can functionally affect the concentration and/or the activity of PON1 [170,179,180,181]. Moreover, they cause a 40-fold variation in enzyme activity between individuals and considerable differences between different populations [182,183,184].

It has been shown that the SNPs of the *PON1* coding region imply the catalytic activity of PON1 in the hydrolysis of specific substrates [143,179,185]. Two main polymorphisms in the coding region of PON1 include a Leu (L)/Met (M) substitution at position 55 (55 Leu (L)/Met (M), and a Gln (Q)/Arg (R) substitution at position 192 has been investigated further in different populations [169,180,186,187,188,189,190,191,192,193,194,195].

The *PON1 55M* isoform is associated with less enzymatic activity than the *PON1 55L* isoform [163]. However, it is unclear whether it is due to the decreased stability of the *PON1 55M* [196] and/or the linkage disequilibrium with the −108T allele [168].

More attention has been paid to the *PON1 Q192R* polymorphism due to the two isoforms, which differ considerably in their affinity for and catalytic activity with many substrates [197,198]. The *PON1 Q192R* polymorphism affects the catalytic efficiency of PON1 toward some of its substrates [178,197,199,200]. For example, the *PON1 192R* isoform hydrolyzes paraoxon (an active metabolite of the parathion) up to six times faster than the 192Q isoform, but some organophosphates, sarin, soman, and lactones are hydrolyzed faster by *PON1 192Q* isoform [199,200]. However, phenylacetate hydrolysis, i.e., AREase activity, is unaffected by Q192R polymorphism [143,177,201,202].

**Table 1:** Paraoxonase gene polymorphisms.

Variation	Allele Change	Residue Change	Rs Number	Location	Ref.
L55M	TTG ⇒ ATG	L [Leu] ⇒ M [Met]	rs854560	Codon	[203]
Q192R	CAA ⇒ CGA	Q [Gln] ⇒ R [Arg]	rs662	Codon	[204]
S23A	TCT ⇒ GCT	S [Ser] ⇒ A [Ala]	rs146211440	Codon	[205]
A201V	GCG ⇒ GTG	A [Ala] ⇒ V [Val]	rs80019660	Codon	[205]
P40L	CCT ⇒ CTT	P [Pro] ⇒ L [Leu]	rs141665531	Codon	[205]
V109I	GTA ⇒ ATA	V [Val] ⇒ I [Ile]	rs61736513	Codon	[205]
C-108T	NA	NA	rs705379	Promoter	[206]
C-107T	NA	NA	Genbank, acc. number AF051133		[168]
C-126G	NA	NA	rs705380	Promoter	[168]
G-162A	NA	NA	rs705381	Promoter	[207]
C-761T	NA	NA	rs3735590	Promoter	[208]
A-824G	NA	NA	Genbank, acc. number AF051133	Promoter	[168]
G-832A	NA	NA	rs854571	Promoter	[207]
G -909C	NA	NA	rs854572	Promoter	[170,206]
A -1074G	NA	NA	rs705379	Promoter	[173,209]
G -1266A	NA	NA	rs76283227	Promoter	[206]
C -1434G	NA	NA	rs705382	Promoter	[206]
A -1741G	NA	NA	rs757158	Promoter	[206]

Reference (Ref).

It has been demonstrated that polymorphisms of the *PON1* gene affect the rate of HDL protection against LDL oxidation [184,210]. However, several studies demonstrate the association between *L55M* and *Q192R* polymorphisms and susceptibility to coronary artery disease [178,211]. However, others have failed to find such an association [178,212,213].

## 6. Transcriptional Regulation of PON1

**Figure 2:**
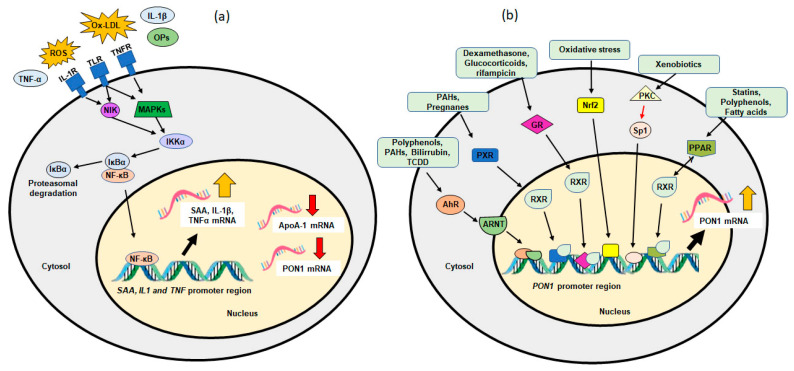
Mechanisms of the transcriptional regulation of *PON1*. (**a**) Regulation by cytokines—the mechanism is still unclear. (**b**) Regulation through nuclear factors activated by endogenous and exogenous factors.

It has also been reported that external and internal elements can influence the activity and expression of PON1; this includes environmental factors, such as pesticides, diet, lifestyle, age, drugs, pathological conditions, and polymorphisms [214,215,216]. Additionally, several transcription factors have been linked to the regulation of *PON1* (Figure 2). Studies have focused on aryl hydrocarbon receptor (AhR), peroxisome proliferator-activated receptors (PPARs), farnesol X receptor, pregnenolone X receptor (PXR), retinoic acid X receptor, glucocorticoid receptor (GR), and vitamin-D receptor (VDR) [217,218,219,220]. However, the mechanisms of how these factors modulate *PON1* remain unclear and should be considered before the realization of its full therapeutic potential [221]. Similarly, its role in different pathological conditions involves oxidative stress and inflammation, since alterations in activity and PON1 levels have been reported in various diseases, such as cardiovascular disease (CVD), metabolic syndrome, Alzheimer’s disease, chronic kidney failure, and chronic liver impairment [136,145].

### 6.1. Endogenous Factors

#### 6.1.1. PON1 and Aging

Studies have shown that low serum activity of PON1 is related to aging and that the increase in age-related oxidative stress could explain, in part, this reduction in PON1 activity of the elderly. Moreover, a decrease in PON1 arylesterase activity in humans during aging shows a correlation with the susceptibility to LDL oxidation [222,223,224]. Therefore, reducing the PON1 activity could negatively affect age-dependent diseases and the aging process rate [225]. In humans, the neonatal PON1 activity is deficient and progressively increases until reaching adult levels at 6 to 15 months. The mechanisms controlling this are unknown [226]. In comparison, Lee et al. [227] suggested that cellular PON1 plays a functional role in cellular senescence and functions as an aging-related protein. Moreover, Seres et al. [222] investigated the PON1 activity and factors influencing its activity as a function of age. They recruited 129 healthy subjects between 22 and 89 years of age. They reported a significant decrease in PON1 activity with age without changing its serum concentration. They also found that HDL from elderly subjects was more susceptible to oxidation than HDL from young subjects measured at a higher lipid peroxidation rate.

#### 6.1.2. PON1 and Polymorphisms

The two most studied polymorphisms, *192QR* and *55LM*, have been associated with PON1 levels and activity. The *55L* allele is associated with higher RNAm and protein serum levels with activity than *55M* [167,228]. Furthermore, low activity has been found in patients with CVD, carrying *55M* alleles [229,230,231]. However, Leviev and James [168] suggested that the *PON1-55LM* effect may be due to linkage to promoter polymorphisms rather than a direct effect of the coding region genotype. Polymorphisms present in the *PON1* promoter region have been investigated for their effect on *PON1* expression levels and are essential for their possible interaction with several transcriptional factors [168,169,170]. Polymorphisms at positions −909 and −162 have a potential nuclear factor 1 (NF-1) transcription factor binding site; meanwhile, −108 is a potential Sp1 binding site, which exerts a two-fold effect on the expression levels of *PON1* and the luciferase activity [170]. Additionally, in people with CVD and diabetes, it is reported that carriers of the −162A allele have higher PON1 activity than −162G [147,209,232].

#### 6.1.3. PON1 and Disease

Oxidant–antioxidant imbalance contributes to the etiology of various diseases, including liver disease, cancer, CVD, kidney disease, Alzheimer’s disease (AD), diabetes mellitus, metabolic syndrome, aging, and some neurological disorders. Additionally, studies suggested the possible role of PON1 in its development [145,233,234].

Decreased PON1 activity has been associated with a high risk of CVD [235]. Similarly, several studies revealed low activity of PON1 in patients with coronary artery disease [236,237], ischemia [238], acute coronary syndrome [203,239] and coronary occlusion [240]. Moreover, the low PON1 activity was inversely related to the severity of coronary artery disease [241]. Additionally, low serum levels of PON1 are an independent risk factor for acute coronary events [235,242,243,244,245].

The level of PON1 as an antioxidant and antiatherogenic enzyme decreases in diabetes mellitus [209,246,247]. PON1 protein and activity reduce the HDLs extracted from patients with diabetes by 2.8 and 1.7 times, respectively, compared to the HDLs extracted from the controls [246]. The patients are more likely to develop heart failure if they have low serum PON1 activity [234]. Additionally, the reduction in PON1 activity with increase in the severity of metabolic abnormalities is a likely consequence of oxidative stress in metabolic syndrome, exceeding the antioxidant capacity of the enzyme. Therefore, PON1 activity is reduced in patients with metabolic syndrome, as demonstrated in a study by Sentí et al. [152]. Reductions in PON1 activity and protein levels in diabetes have been attributed to different mechanisms: (a) reductions in apolipoproteins in HDL may affect the separation of PON1 from HDL and its subsequent instability, (b) the increase in oxidized lipids caused by oxidative stress may contribute to the inactivation of PON1 [246], (c) increased triglycerides in HDL may affect PON1 activity and (d) increases in blood glucose may reduce PON1 activity through protein glycation, including PON1 [248,249].

Additionally, the liver plays a critical role in synthesizing serum PON1, with the gene expression confined only to the liver. Therefore, any injury could influence its status [250]. Nonalcoholic fatty liver disease (NAFLD) is receiving significant attention as a common cause of chronic liver disease. Additionally, nonalcoholic steatohepatitis (NASH), an advanced form of NAFLD, can progress to end-stage liver disorders, including liver cirrhosis and hepatocellular carcinoma. Furthermore, NAFLD increases the risk of other comorbidities, including cardiometabolic diseases. Oxidative stress is recognized as a causative factor of NAFLD, resulting in inflammation and fibrosis in the liver. Although various molecules, lipids, and proteins are oxidized in that process, the detailed mechanisms remain partially elucidated [251]. As PON1 is mainly produced in the liver, it may become an essential helpful end research target in liver disease. Moreover, human studies demonstrated that the PON1 activity is low in patients with chronic liver disease, including alcoholic liver injury [252,253] and viral hepatitis [250]. A recent experimental study also demonstrated low PON1 activity in rats with NAFLD [254].

Oxidative stress, such as LDL oxidation, is thought to be an important mechanism in many degenerative diseases, including atherosclerosis, diabetes, and AD [255]. Furthermore, Saeidi et al. [256] showed that PON1 activity is significantly lower in Alzheimer’s patients than controls, showing low arylesterase activity as a risk factor for this disease. Meanwhile, other studies conducted on the Chinese population did not find any difference in enzyme activity of Alzheimer´s patients compared with controls [257,258]. However, a recent study demonstrates that PON-arylesterase activity significantly decreases in Alzheimer´s patients compared to in controls [259].

In epidemiological studies, confounding variables such as environmental and physiological factors should be obtained before analysis. Sex is a biological factor that impacts PON1 expression and activity. In humans, both arylesterase and lactonase activity is increased in women compared to men. The difference was independent of age, HDL-c, and smoking [260]. However, sex differences in humans can be minimized by the genetic diversity of the populations, since several epidemiological studies found significant differences in PON1 activities between women and men [261,262,263]. Additionally, in animal studies, female rats showed higher PON1 activity than male rats, showing consistency with the gender difference in PON1 activity [264,265].

### 6.2. Exogenous Factors

It has been shown that various non-genetic factors can modulate the activity and expression of PON1 through different mechanisms partially understood, involving nuclear receptors, at least, at the transcriptional level. The most prominent factors are diet, drugs, and environmental pollutants [136,216,266].

#### 6.2.1. Dietary Factors and PON1

The consumption of fruits, vegetables, and red wine is associated with benefits against oxidative stress diseases, major due to polyphenols. Studies have shown that fruit juices and red wine polyphenols can increase the activity of PON1 both in mice and in humans [267,268]. Quercetin, aspirin, naringenin, flavone, resveratrol, and polycyclic aromatic hydrocarbons act through AhR, since the *PON1* promoter region contains a nonclassical core ARE-like motif between positions −126 and −106, as reported by Gouédard et al. [269]. Additionally, several studies have suggested that PON1 is regulated by a mechanism that involves the AhR activation [220,270,271,272,273]. Additionally, treating HepG2 and HuH7 hepatocytes with berberine increased *PON1* expression at the transcriptional level via a JNK/c-Jun signaling pathway [219]. The Mediterranean diet has also been shown to exert a beneficial effect on PON1 activity. Extra virgin olive oil has effectively increased PON1 activity, and oleic acid enrichment of phospholipids present in high-density lipoproteins favored the activity and increased hepatic PON1 mRNA and protein expressions induced by minor components present in this oil. Other Mediterranean diet constituents, such as nuts, fruits, vegetables, and pomegranate, have modulated PON1 activity [274].

#### 6.2.2. Drugs and PON1

It has been demonstrated that statins, glucose, and quercetin induce *PON1* expression through the cholesterol transcriptional regulator (Sterol response element binding protein 2 (SREBP2)) and Sp1 binding to the *PON1* promoter region. The presence of an important SREBP2 (bp –104 to –95) in the proximal PON1 promoter region suggests an important PON1–lipoprotein relationship. Furthermore, SREBP-2 increased the *PON1* promoter activity in a dose-dependent manner, indicating an additional lipid-related mechanism of PON1 activation [162]. Additionally, hypolipidemic drugs upregulate *PON1* synthesis through PPAR activation [29]. However, activations of PPARα and the possible induction of the *PON1* gene are controversial; the increase in *PON1* expression by fibrates suggests it does not involve this nuclear receptor. However, in vivo treatment with fenofibrate increased *PON1* expression, but the in vitro result of this induction is unclear [129,153], since it was found to be a potential binding site of PPARα on the *PON1* promoter region [29]. Similarly, several studies suggested that statins such as simvastatin, atorvastatin, and rosuvastatin, and polyphenols obtained from plants increase the *PON1* expression by the PPARγ activation [275,276,277,278,279]. Additionally, polyphenols from pomegranate juice increased *PON1* expression via the PPARγ-PKA-cAMP pathway in Huh7 cells. Additionally, a urokinase-type plasminogen activator (uPA) was observed that decreased *PON1* expression by inactivating PPARγ, and this mechanism depended on uPA-mediated mitogen-activated protein kinase activity [280]. However, treatment with dexamethasone in human hepatoma cells increased *PON1* expression in a time-dependent GR activation; the interactions of the response element–GR ligand complex in the *PON1* promoter region were confirmed by chromatin immunoprecipitation between −95 and −608 base pairs of the promoter region [29].

#### 6.2.3. Environmental Pollutants and PON1

The study of the effect of environmental pollutants on PON1 has been focused mainly on pesticides. It has been reported that putative binding sites for PXR exist in the *PON1* promoter region [281], and studies have shown that organochloride insecticides and dieldrin can activate PXR-regulated regulated gene expression in rat cells [282]. However, PXR in *PON1* expression is limited, and the mechanism involved in *PON1* regulation is still unclear. The protective effect of PON1 on dichlorvos-induced liver oxidative stress injury in mice has been studied. The upregulation in *PON1* reduced malondialdehyde content, enhanced superoxide dismutase (SOD) and catalase (CAT) activities, increased GSH content, and may have also upregulated Nrf2 mRNA expression [283]. This suggests the possible role of Nrf2 in *PON1* expression, since Wagner et al. [284] identified three putative binding sites on the *PON1* promoter region. Moreover, hepatocytes incubated with roasted curcumin or 4-vinyl guaiacol showed enhanced transactivation of Nrf2 and the induction of *PON1* [285]. However, the mechanisms underlying the *PON1* transcriptional regulation through Nrf2 are not entirely understood. Moreover, several studies have reported the decrease in PON1 activity due to oxidative stress-mediated inflammatory reactions in organophosphate (OP) exposure [286,287,288]. Additionally, it has been reported that higher proinflammatory cytokines and lower PON1 in OP exposed individuals [289]. Low activity of PON1 is considered a biomarker of susceptibility to organophosphate compounds due to the ability of PON1 to modulate the toxicity of organophosphates [143].

### 6.3. Epigenetics

With the current breakthrough in omics platforms, environmental exposure in utero and during the early stages of development can cause permanent changes through epigenetic programming, leading to chronic degenerative diseases as severe as cancer.

Therefore, we consider the definition of current epigenetics, involving molecular modifications in DNA that can regulate gene activity independent of the DNA sequence, whose changes are, in turn, mitotically stable [290]. Previously, the study of epigenetics focused on how the phenotype is associated with changes at the chemical, structural, and DNA regulatory levels; for their part, environmental stimuli can affect the epigenetic regulation by influencing the predisposition to various diseases [291]. The study of mechanisms of epigenetic regulation through gene expression includes DNA methylation, histone modifications, the study of non-coding RNAs [292], and genome-wide DNA methylation profiling.

The relationship between the social environment and health has been established in epidemiology. The social (e.g., neighborhood in which we live, exposure to pollutants, among others) and physical (e.g., body mass index, age, among others) dimensions are involved in this context concerning the environment; factors that interact together regarding an organism, determining its development and survival [293,294]. Furthermore, some pollutants can alter the biological systems within the social environment through different biological processes. Recent findings in molecular epidemiology have enhanced elucidating some of the possible mechanisms caused by environmental stimuli, in conjunction with susceptibility to exposure, that influence the disease’s development in the use/study of PON1 (Table 2).

PON1 has been cataloged as a model for integrating genetic and epigenetic data due to its multifunctional enzyme involved in oxidative defense, with its genetic variants and lower levels associated with adverse health effects, such as alterations in neurodevelopment and at the cardiometabolic level [295].

Age and different exogenous and endogenous factors, and genetic context, are potential factors associated with PON1 variability and susceptibility through epigenetic regulation and the study of different mechanisms in this area; the relationship with the presence of different diseases can be explained [296]. The *PON1* gene is highly polymorphic and more than 400 SNPs identified in the coding region, introns, and regulatory region have been reported. However, many of these SNPs remain unidentified, but the SNPs studied and identified have presented a direct relationship with the activities of PON1 [144].

The *PON1* gene is a member of a group of genes that include *PON1*, *PON2*, and *PON3* and remain adjacent to each other located on chromosome 7. Specifically, the *PON1* promoter region has a CpG island comprising 19 CpG sites, and a second island mapped near exon 7 contains eight CpG sites. Overall, 287 CpG sites were reported in *PON1* [296].

So far, most of the studies on epigenetic modifications in the *PON1* gene have focused on the varying DNA methylation levels. Changes in methylation profiles, specifically at these sites, may lead to potential effects at the gene expression level due to the specific binding of transcription factors at these CpG sites; however, epigenetically, data on the methylation of these sites are scarce [296]. Hypo- and hyper-methylation at CpG sites along the *PON1* have been evidenced in the promoter and the coding regions of this gene in populations exposed to several toxic compounds, such as heavy metals and pesticides, and in populations diagnosed with the development of different diseases, including metabolic syndrome, ischemic stroke, and coronary artery disease, among others [297,298,299].

**Table 2:** Epigenetic regulation and *PON1* studies.

Subjects DNA Methylation	*n*	Analytical	Main Findings	Ref.
Obese adults with metabolic syndrome	47	Infinium Human Methylation 450 K BeadChip	A significant inverse correlation was found between the PON1 methylation in the CpG 1 (Chr position = 7:94,953,956), CpG 2 (Chr position = 7:94,954,059), CpG 3 (Chr position = 7:94,954,144) and CpG 4 (Chr position = 7:94,954,202) sites with the enzymatic AREase activity.	[297]
Age [mean ± SD] = 47 ± 10 years.			Negative correlations were found between the selected antioxidants (vitamin C, total tocopherols, and lycopene) with the percentage of methylation of the different PON1 gene CpG sites.	
Patients with parenchymal ischemic stroke.		Pyrosequencing	No interaction was observed between the body composition and stroke diagnosis criteria for either of the analyzed CpG sites of the PON1 gene. No relevant changes were observed in the PON1 total methylation patterns considering stroke or obesity conditions.	[298]
Age [mean ± SD] = 70 ± 12 years.			The CpGs at +15 and +241 bp in the PON1 promoter were related to weight, waist circumference, and energy intake in the group of patients without stoke, and an interaction was observed between the energy intake and total PON1 promoter methylation in the prediction of stroke condition (*p* = 0.017).	
Children of a farmworker community (pesticide exposure).	449	Infinium Human Methylation 450 K BeadChip	Among sites in methylation block 1 (CpG sites 5, 8, 11, and 13), a separation by genotype was observed, providing evidence of allele-specific methylation.	[295]
Newborns and 9-year-old children			Strongly positive associations were found between PON1 −108 T alleles and methylation levels, particularly those in methylation block 1. Methylation at individual Block 1 CpG sites was significantly associated with AREase activity.	
Newborns with prenatal mercury (Hg) exposure.	321	Infinium Human Methylation 450 K BeadChip	A DMR covering 9 CpG sites of Chr 7 in the PON1 gene was hypomethylated by prenatal Hg exposure among boys. In early childhood, a doubling in prenatal Hg concentration was associated with a 4.6% decrease in methylation levels of the DMR in PON1. Two CpG sites (cg07404485 and cg05342682) in the PON1 DMR located in the body of the gene and in the north shore region of a CpG island had the strongest association with expression evaluated in cord blood samples.	[300]
Children in early childhood (2.9–4.9 years) and mid-childhood (6.7–10.5 years).				
Children born by female greenhouse workers (pesticide exposure).	48	Infinium Human Methylation 450 K BeadChip array and pyrosequencing	Considering the effect modification by PON1 Q192R genotype, 767 significantly DMPs were identified, of which 128 were hypermethylated and 639 were hypomethylated. 5002 significant DMRs were identified, of which 2264 were hypermethylated and 2738 hypomethylated in the exposed PON1 192R carrier group compared to the other groups. The pyrosequencing methylation values were significantly more highly methylated in exposed children compared with the unexposed group carrying the PON1 192R-allele for most CpG sites.	[301]
Age: 6–11 years				
Patients with coronary artery disease.	484	Pyrosequencing	The PON1 −162 A>G genotype may significantly influence the methylation level at PON1 CpG site −162. Five CpG sites (positions −184, −170, −163, −161, and −142) exhibited hypomethylation in association with the occurrence of bleeding. Multivariate logistic regression analysis showed that methylation at PON1 site −161 and the use of angiotensin-converting enzyme inhibitors were associated with a decreased risk of bleeding events.	[299]
Age: 26–80 years				
Patients with overweight or obesity.	790	Pyrosequencing	Similar patterns between ten of the distinct CpGs for genetics, expression and activity across the 11 CpG sites were evaluated. Methylation levels varied between 6% and 60%; lower methylation levels were observed at CpG site −108 (10.4%) compared with the average promoter methylation (CpGmean = 27.9%). A significant association between average promoter hypermethylation and reduced expression on PON1.	[302]
Age: 18–74 years				
Histone modification				
In vivo study	10	ChIP analysis	At the promoter and coding regions, H3Ac was not significantly different between control and high fat offspring in males but slightly decreased in females.	[303]
Rats with two dietary treatments: control and high fat groups.			At the promoter, H4Ac and H3K4Me2 were significantly higher in both male and female high fat offspring compared with the control group.	
Modifications in miRNA expression				
Patients with ischemic stroke. Age [mean ± SD] = 62.23 ± 11.72 years.	2228	MicroRNA expression profiling microarrays	miR-616 that binds to PON1 displayed increased expression in vascular smooth muscle cells treated with oxidized low-density lipoprotein and lipopolysaccharide. They found that miR-616 negatively regulates the expression of *PON1*.	[208]
Patients with coronary artery disease. Age: 24–79 years	111	Pathway-focused Human CVD miScript miRNA PCR array	Bivariate parametric correlation analysis showed that the serum PON1 activity correlated negatively with miR-486, miR-92a, and miR-122. The logistic regression model including miR-92a and miR-486 serum levels with adjustment for age, gender, serum lipids and apolipoproteins levels and PON1 activity as covariates resulted in a significant designation of vulnerable coronary artery disease patients with an accuracy of 84%.	[304]
Patients diagnosed with chronic obstructive pulmonary disease.	292	Quantitative real-time polymerase chain reaction	miR-616 regulates the PON1 expression in primary hepatocytes genotyped as CC.	[305]
Age [mean ± SD] = 60.22 ± 8.24 years.			miR-616 down-regulated the expression of PON1 and inhibited the PON1 activity in primary hepatocytes.	
Patients with calcific aortic valve stenosis	459	Quantitative real-time polymerase chain reaction	The loci rs3735590 of *PON1* 3′UTR contains the binding site of miR-616. As a negative regulator of PON1, upregulation of miR-616 inhibited the expression of this gene, by binding to the 3′UTR of the mRNA.	[306]
Age [mean ± SD] = 72.2 ± 9.6 years.				

Chr: chromosome, SD: standard deviation; AREase: arylesterase; bp: base pairs; DMPs: differentially methylated positions; DMRs: differentially methylated regions; ChIP: chromatin immunoprecipitation; H3Ac: acetylated histone H3; H4Ac: acetylated histone H4; and H3K4Me2: dimethylated histone H3 at lysine residue 4.

Regarding epigenome studies, differentially methylated regions (DMRs) and differentially methylated positions (DMPs) have been associated with the presence of specific genotypes of *PON1* and the development of cardiovascular disease. Interestingly, it has been identified that most of these DMPs are located in the promoter region, CpG islands, and in the transcription factor-binding sites, suggesting a possible direct link with gene expression and, in turn, some genes that relate to these DMPs have been associated with several neuroendocrine signaling pathways [301]. 

Additionally, regarding histone modifications, there have been no population studies focused on evaluating the implications of this epigenetic modification on the expression of *PON1*; however, only one study has evaluated, through an obesity-resistant rat model, the implications of the epigenetic regulation by histone methylation on the expression of this gene. Results at the promoter region show that acetylated histone H4 (H4Ac) and dimethylated histone H3 at lysine residue 4 (H3K4Me2) marks were significantly higher in the high-fat group than the control group [303], suggesting that the epigenetic regulation across of histone modifications represents a critical mechanism of *PON1* regulation. However, longitudinal studies are necessary to extend the study of these modifications and their involvement in the possible development of diseases attributed to the loss of *PON1* regulation.

However, regarding the expression of miRNAs assessed by in silico analyses, several miRNAs have been identified, including miR-616, miR-19a, miR-26a, miR-505, miR-495, miR-153, and miR-185, involved in the negative regulation of PON1 [295]. Moreover, negative correlations have been found between miRNA levels (e.g., miR-92a, miR-486, and miR-122) with the PON1 enzymatic activity and the development of cardiovascular diseases [304]. Recent studies have revealed the specific importance of miR-616 in the direct regulation of PON1 expression. The binding site of this miRNA is located on SNP rs3735590C/T, a polymorphism associated with the development of coronary artery disease. Additionally, it has been revealed that miRNA plays a fundamental role in susceptibility to ischemic stroke and atherosclerosis [307].

#### From Epigenetic Regulation of DNA to Cancer

Based on DNA methylation, the epigenetic markings on this regulation mechanism in the mammalian genome indicate that about 70% of CpG dinucleotides are methylated, representing the possible silencing of non-coding DNA regions, including introns, repetitive elements, and potentially active transposable elements [144]. The specific association of PON1 AREase activity with the presence at the cardiovascular alteration level can be explained through the methylation study of CpG sites in the promoter region of the *PON1* gene. Thus, it has been evidenced in an inverse association between the methylation of CpG sites of the promoter of *PON1* with the activity AREase in adults with dietary restriction [297]. This condition could be associated with the development of different types of cancer associated with a lack of nutrients in the diet and potent antioxidants in food. Based on studies related to the presence of cancer and the epigenetic regulation of *PON1*, the corresponding data remain scarce in the literature; however, the *PON2* and *PON3* genes have been studied, focusing on the levels of the methylation profile of these genes (Table 3). The epigenetic regulation of *PON1* is directly linked to the presence of cardiometabolic alterations and those involving oxidative stress processes that can also be related to the presence of cancer in different tissues [307].

Regarding non-coding RNAs (ncRNAs), Chen et al. [104] investigated the effect of ncRNA on the lactonase activity of PON1. It was shown that the specifically low levels of Linc-OIP5 correspond to lower levels of PON1 expression. In contrast, the high levels in the expression of miR-616 had an opposite effect and decreased the expression of PON1 and lactonase activity [104]. Lactonase activity can be a fundamental tool for explaining the presence of cancer coupled with inter-individual variability in human populations. Epidemiological studies have revealed a relationship between *PON1* polymorphisms implicated in the different types of cancer development and reduction in serum PON1 activities, such as lung, colorectal, prostate, and breast cancer, among others [157,308,309,310,311]. Different molecular mechanisms have been proposed between cancer development and serum activity of PON1 reduction, among which are the down-regulation of *PON1* synthesis by the cytokines TNFα and IL-1β, the inhibition of PON1 activity by oxidized phospholipids, and alteration in compositional modifications of PON1 protein (e.g., alterations of protein glycosylation and fucosylation patterns) [312].

**Table 3:** Epigenetic modifications focused on *PON1* studies and cancer development.

Subjects PON1	*n*	Epigenetic Modification	Analytical Method	Main Findings	Ref.
Patients diagnosed with colorectal cancer (CRC).	30	Histone modification	Treatment with the lysine developer and analysis using a microplate reader	The results indicate that the CRC group showed a statistically significant increase in histone deacetylase activity in comparison with the control group. CRC group showed an elevation of oxidative stress landmarks, histone deacetylase activity and a significant decrease in the level of PON1 activity. Correlation matrix showed a positive correlation between advanced oxidation protein products concentration and histone deacetylase activity; while negative correlations were found between PON1 levels and histone deacetylase activity; as well as between PON1 levels and advanced oxidation protein products concentration. This study suggested that histone acetylation/deacetylation process could have an important role in cancer cell development and the early estimation of histone deacetylase activity in cancer cell may give early diagnostic method.	[313]
Patients with renal cell carcinoma (RCC).	15	DNA methylation	Methylation-specific polymerase chain reaction	Of the 15 RCC tumor tissues, 12 had high methylation of *PON1* gene. *PON1* showed hypermethylation in kidney renal papillary cell carcinoma compared with normal tissue. The 7 differentially methylated imprinted sites including cg01874867, cg04155289, cg05342682, cg07404485, cg17330251, cg19678392, and cg21856205 presented higher methylation levels in the RCC than in the normal cells. Methylation analysis of the paired tumor and normal tissues showed 50 most differentially methylated genes. Kaplan–Meier analysis showed that hypermethylated *PON1* had shorter lifespan generally. Hypermethylation of *PON1* was present in the progression of RCC.	[314]

RCC: Renal Cell Carcinoma.

Nowadays, the variation of DNA methylation levels of *PON1* has been linked to the development of diseases, such as vascular dementia and cardiovascular diseases, among others. Nevertheless, information about the *PON1* gene and DNA methylation in cancer development is still scarce. A study in renal cell carcinoma (RCC) found second differentially methylated imprinted sites related to RCC’s higher methylation levels than normal cells. Of the 15 RCC tumor tissues evaluated, 12 had high methylation of *PON1*. Additionally, this study demonstrated that the demethylation of *PON1* inhibited the migration, invasion, and proliferation of renal cancer cells and arrested more cells in the G0/G1 phase [314]. These findings expose a research gap on the DNA methylation of *PON1*, which could be used as a targeted biomarker in treating cancers, such as RCC. Moreover, in colorectal cancer (CRC), patients were found with elevated oxidative stress landmarks (DNA damage and advanced oxidation protein products), a decrease in the level of PON1 activity, and an increase in histone deacetylase activity, suggesting the crucial influence of histone deacetylase activity on PON1 in cancer development [313].

## 7. PON1 and Cancer

Several studies have demonstrated the relationship between oxidative stress and the development of human diseases among these cancers. Recently, many studies have focused on the relationship between PON1 and cancer. In this regard, some studies have reported low expression and activity of PON1 (PONase, AREase, LACase) in patients with different types cancer such as cancer blander [315], gastrointestinal cancer [308,316,317], breast cancer [308,318], prostate cancer [310,318], lung cancer [157,308,318], non-Hodgkin lymphoma [318], and central nervous system tumors [319] (Table 4). As observed in Table 4, the variability in PON1 activity is extensive. Low PON1 activity affects its intrinsic functions and increases oxidative stress [252,253,254,255,256,257,258], suggesting a worse prognosis in patients with cancer [320]. Huang et al. [321] proposed that serum PON1 levels could be used as a biomarker for microvascular invasion. They found that *PON1* expression is inversely associated with a degree of vascular invasion in hepatocarcinoma cells.

Additionally, polymorphism genetics of the *PON1* gene have been implicated in cancer development. The polymorphism of *PON1* most studied is *PON1 Q192R* because the two isoforms differ considerably in their affinity for and catalytic activity with several substrates. This polymorphism has been related to bladder cancer, renal cancer, prostate cancer, and lymphoma (Table 5). However, the meta-analysis study conducted by Zhag et al. [322] showed that the *PON1-192R* had reduced risk in the development of brain cancer and breast cancer. Concerning *PON1 L55M*, it is also associated with a higher risk of prostate cancer [323] (Table 5). More recently, in the meta-analysis study conducted by Liu et al. [324], only 40 studies of 233 were included, and the polymorphism *PON1 L55M* was significantly associated with hematological tumors and breast cancer. Therefore, these authors suggest this polymorphism as a biomarker to susceptibility to identify an individual with a risk of hematological tumors and breast cancer.

Since we did not find information on other *PON1* polymorphisms regarding cancer association, more studies are required to establish the other *PON1* polymorphisms’ contribution to cancer development.

Based on the studies, it is evident that PON1 plays a vital role in cancer. However, it is important to account for the cancer type, ethnicity, gene interactions, and several factors contributing to cancer development. Additionally, the extensively signal transduction pathways involved in the modulation of *PON1* and the relationship between PKC and MAPK/ERK pathways activated by growth factors to regulate cell growth and differentiation, apoptosis, and angiogenesis [214].

## 8. Conclusions

This review involves the role of PON1 and cancer. Undoubtedly, the gene expression of PON1 will remain challenging to study and therefore, more studies at different levels of scientific research are required for the complete elucidation of the role of this enzyme and its specific relationship with cancer. Therefore, targeting PON1, redox-sensitive pathways and transcription factors promise disease prevention, including cancer.

## Figures and Tables

**Table 4:** Human studies and PON activity of patients with serval type of cancer.

Cancer Type	Type of Study Control vs. Case	PON1 Activity	Reference
Bladder cancer	Control (*n* = 57)	PONase (U/L):137.6 ± 53.3	[315]
		AREase (U/L): 168.82 ± 37.4	
	Case (*n* = 56)	PONase (U/L):103.35 ± 41.1	
		AREase (U/L): 131.8 ± 39.9	
Colorectal cancer	Control (*n* = 25)	PONase (U/mL):124.8 ± 21.7	[308]
		AREase (U/mL): 98.5 ± 18.8	
	Case (*n* = 40)	PONase (U/mL):65.9 ± 22.5	
		AREase (U/mL): 57.5 ± 13.34	
	Control (*n* = 39)	PONase (U/L):230.5 ± 97.9	[316]
		AREase (k/mL): 230.7 ± 53.8	
	Case (*n* = 40)	PONase (U/L):128.2 ± 63.2	
		AREase (k/mL): 150.6 ± 49.2	
	Control (*n* = 80)	PONase (U/mL):394.1 ± 83.4	[317]
		AREase (U/mL): 228.4 ± 83.4	
	Case (*n* = 50)	PONase (U/mL): 272.6 ± 118.8	
		AREase (U/mL): 197.3 ± 72.1	
Breast cancer	Control (*n* = 25)	PONase (U/mL):124.8 ± 21.7	[308]
		AREase (U/mL): 98.5 ± 18.8	
	Case (*n* = 25)	PONase (U/mL):42.9 ± 7.9	
		AREase (U/mL): 54.5 ± 11.5	
	Control (*n* = 50)	PONase (U/mL):95.0 ± 30.3	[318]
		AREase (U/mL): 52.1 ± 11.9	
	Case (*n* = 50)	PONase (U/mL):65.0 ± 28.2	
		AREase (U/mL): 29.0 ± 03.02	
Prostate cancer	Control (*n* = 20)	PONase (U/mL):92.1 ± 36.4	[310]
		AREase (U/mL): 51.1 ± 13.9	
	Case (*n* = 25)	PONase (U/mL):67.2 ± 6.2	
		AREase(U/mL): 31.0 ± 14.6	
	Control (*n* = 40)	PONase (U/L): 76.5 ± 46.5	[318]
		AREase (kU/L): 135.5 ± 65.7	
	Case (*n* = 23)	PONase (U/L):103.8 ± 64.7	
		AREase (kU/L): 136.7 ± 59.9	
Lung cancer	Control (*n* = 25)	PONase (U/mL):124.8 ± 21.7	[157]
		AREase (U/mL): 98.5 ± 18.8	
	Case (*n* = 45)	PONase (U/mL):67.6 ± 22.0	
		AREase (U/mL): 65.6 ± 27.1	
	Control (*n* = 39)	PONase (U/mL): 395.8 ± 116.6	[308]
		AREase (U/mL): 167.7 ± 45	
	Case (*n* = 39)	PONase (U/mL):252.7 ± 104.4	
		AREase (U/mL): 137.4 ± 57.6	
	Control (*n* = 12)	PONase (U/mL): 94.6 ± 24.2	[318]
		AREase (U/mL): 54.7 ± 05.3	
	Case (*n* = 12)	PONase (U/mL):70.2 ± 12.4	
		AREase (U/mL): 36.4 ± 02.3	
Non-Hodgkin lymphoma	Control (*n* = 10)	PONase (U/mL): 89.3 ± 32.4	[318]
		AREase (U/mL): 51.1 ± 12.7	
	Case (*n* = 10)	PONase (U/mL):62.2 ± 0.7	
		AREase (U/mL): 37.0 ± 0.6	
Central Nervous System Cancer	Control (*n* = 50)	PONase (U/L): 218.8 ± 144.8	[319]
	Case (*n* = 42)	PONase (U/L):73.2 ± 62.0	
	Control (*n* = 25)	PONase (U/L): 131.1 ± 14.0	
	Case (*n* = 25)	PONase (U/L):70.6 ± 17.0	

PON activity: paraoxonase (PONase). Arylesterase (AREase).

**Table 5:** Association between *PON1* polymorphisms and cancer susceptibility.

SNP	Genotype	Cancer Type	Population	Reference
*L55M*	*55M*	Breast cancer	Caucasian	[325]
*L55M* *Q192R*	*55M* *192QQ*	Breast cancer	Caucasian Italian	[31]
*L55M*	*55M*	Breast cancer	Egyptian	[326]
*L55M*	*55M*	Breast cancer	Malaysian	[327]
*Q192R*	*192QQ*	Lymphoma	Spain	[328]
*Q192R*	*192QQ*	Glioma	Chinese	[329]
*L55M*	*55M*	Leukemia	Brazilian	[330]
*Q192R*	*192QQ*	Lymphohaematopoietic cancer	Greeks	[331]
*Q192R*	*192QQ*	Lung cancer	Turkish	[332]
*L55M* *Q192R*	*55M* *192QQ*	Ovarian cancer	Hawaii	[333]
*Q192R*	*192QQ*	Osteosarcoma	Caucasian Turkish	[334]
*L55M* *Q192R*	*55M* *192QQ*	Brain tumor	Spain	[335]
*Q192R*	*192RR*	Bladder cancer	Turkish	[336]
*Q192R*	*QQ*	Hodgkin’s lymphoma	Caucasian	[337]
*L55M*	*55M*	Breast cancer	Caucasian Italian	[31]
*Q192R*	*RR*	Brain tumor	Turkish	[319]
*Q192R*	*192RR*	Ovarian cancer	Turkish	[338]
*C108T*		Brain tumor	Mixed	[339]
